# Studying Staphylococcal Leukocidins: A Challenging Endeavor

**DOI:** 10.3389/fmicb.2020.00611

**Published:** 2020-04-15

**Authors:** Angelino T. Tromp, Jos A. G. van Strijp

**Affiliations:** Department of Medical Microbiology, University Medical Center Utrecht, Utrecht, Netherlands

**Keywords:** *S. aureus*, toxin, leukocidin, immune evasion, pore-formation, *in vivo*, CA-MRSA, bacterial pathogenesis

## Abstract

*Staphylococcus aureus* is a well-known colonizer of the human skin and nose, but also a human pathogen that causes a wide spectrum of diseases. It is well established that *S. aureus* secretes an arsenal of virulence factors that have evolved to circumvent the human immune system. A major group of *S. aureus* virulence factors is the bi-component β-barrel pore-forming toxins, also known as leukocidins. These pore-forming toxins target specific cells of the innate and adaptive immune system by interacting with specific receptors expressed on the cell membrane. Even though still heavily debated, clinical and epidemiological studies suggest the involvement of one of the bi-component toxin, Panton-Valentine Leukocidin (PVL), as an important factor contributing to the epidemic spread and increased virulence of CA-MRSA strains. However, the host- and cell-specificity of PVL and other leukocidins, and the lack of adequate *in vivo* models, fuels the controversy and impairs the appropriate assessment of their role in *S. aureus* pathophysiology. Currently, the mechanisms of pore-formation and the contribution of PVL and other leukocidins to *S. aureus* pathophysiology are incompletely understood. This review summarizes our current understanding of leukocidin pore-formation, knowledge gaps, and highlights recent findings identifying novel host-factors involved in the toxin-host interface. As a result, this review furthers emphasizes the complexity behind *S. aureus* leukocidin cytotoxicity and the challenges associated in the quest to study and understand these major virulence factors.

## Introduction

*Staphylococcus aureus* is a major bacterial pathogen in humans that, combined with the acquisition of antibiotic resistance, is of serious concern to public health (Katayama et al., 2000; Whitby et al., 2001; Thwaites et al., 2011; David and Daum, 2017). Neutrophils are the first to arrive at the site of infection and subsequently phagocytose and kill *S. aureus* (Rigby and DeLeo, 2012; Spaan et al., 2013b; van Kessel et al., 2014). Neutrophils play a crucial role in the containment and clearance of *S. aureus* (Rigby and DeLeo, 2012; Spaan et al., 2013b; van Kessel et al., 2014). It is therefore not surprising that many *S. aureus* secreted proteins inhibit phagocytosis by targeting neutrophils. It has been evident for more than a century that *S. aureus* secretes proteins that interact and kill leukocytes (Van De Velde, 1894; Van De Velde and Denys, 1895; Panton and Valentine, 1932). It took almost 7 decades to attribute this leukocidal activity to cytolytic toxins secreted by *S. aureus* (Woodin, 1959, 1960; Woodin and Wieneke, 1963). *S. aureus* produces cytolytic peptides known as Phenol Soluble Modulins (PSMs), and β-barrel pore forming toxins, such as the single-component α-Hemolysin (Hla) and multiple bi-component toxins that target the cell membrane resulting in the lysis of host immune cells (Valeva et al., 1997; Wang et al., 2007; DuMont and Torres, 2014). It is now clear that all human *S. aureus* isolates are able to produce potent bi-component toxins, better known now as leukocidins, that target and lyse phagocytes. Even though still heavily debated, clinical and epidemiological studies suggest a paradoxal involvement of the bi-component toxin Panton-Valentine Leukocidin (PVL) as an important factor contributing to the epidemic spread and increased virulence of CA-MRSA strains (Lina et al., 1999; Alonzo and Torres, 2014; Li et al., 2016; Wang et al., 2018). However, the mechanisms of pore formation and the contribution of PVL and other leukocidins to *S. aureus* pathophysiology are incompletely understood. It was long assumed that leukocidins interact with lipid constituents on target cells (Noda et al., 1980; Ozawa et al., 1994; DuMont and Torres, 2014). However, lipids alone could not explain the apparent host- and cell-tropism of leukocidins, indicating that there are additional host-factors involved in leukocidin cytotoxicity and phagocyte targeting (Panton and Valentine, 1932; Colin et al., 1994; Gauduchon et al., 2001; Potrich et al., 2009; DuMont and Torres, 2014). The first major breakthrough came when a specific proteinaceous receptor, the transmembrane metalloprotease ADAM10, was identified for Hla (Wilke and Bubeck Wardenburg, 2010). This catalyzed the identification of specific receptors for all leukocidins (Alonzo et al., 2013; Reyes-Robles et al., 2013; Spaan et al., 2013a, 2014, 2015a,b), clarifying the observed species- and cell-specific toxicity of *S. aureus* leukocidins. Human *S. aureus* isolates secrete up to five different leukocidins that target phagocytes; PVL, γ-haemolysin AB (HlgAB) and CB (HlgCB), leukocidin ED (LukED) and leukocidin GH (LukGH, also known as LukAB) (Ventura et al., 2010; Vandenesch et al., 2012; Spaan et al., 2017). In addition, *S. aureus* strains associated with zoonotic infections have been described to secrete leukocidin MF’(LukMF’) and leukocidin PQ (LukPQ) (Vrieling et al., 2016; Koop et al., 2017). Murine infection models, to an extent, have proven useful in understanding the role of these leukocidins in *S. aureus* pathogenesis (Rauch et al., 2012; Parker, 2017). The leukocidin LukED targets cells of the adaptive immunity via CCR5 and is compatible with murine CCR5 (Alonzo et al., 2013). This made it possible to show the contribution of LukED as it kills cells of the adaptive immune system in mice *in vivo* (Alonzo et al., 2013). In addition, LukED targets neutrophils, monocytes and NK-cells via CXCR1 and CXCR2, which also promote *S. aureus* pathogenesis in mice *in vivo* (Reyes-Robles et al., 2013). Another leukocidin, HlgAB, targets CCR2, CXCR1 and CXCR2. HlgAB interacts with murine inflammatory macrophages via CCR2, as shown in a murine peritonitis model (Spaan et al., 2014). However, due to incompatibility between HlgAB and murine CXCR2, current mouse models are not suitable to fully comprehend the extent of HlgAB contribution to *S. aureus* pathogenesis *in vivo* (Spaan et al., 2014). Other leukocidins that exert a narrow host-specificity, such as HlgCB and PVL that interact with human C5aR1 (Spaan et al., 2013a, 2015b), have been difficult to study *in vivo*. Murine models have failed to demonstrate a role for PVL in *S. aureus* pathogenesis *in vivo*, as murine neutrophils are resistant to PVL (Voyich et al., 2006; Bubeck Wardenburg et al., 2008; Loffler et al., 2010). Other studies addressing the role of *S. aureus* and PVL in non-human primates (Olsen et al., 2010; Kobayashi et al., 2013) also failed to observe a PVL contribution in *S. aureus* pathophysiology, likely as non-human primate neutrophils are also resistant to PVL (Loffler et al., 2010; Spaan et al., 2013a). In contrast, rabbit neutrophils are at least as susceptible to PVL toxicity as human neutrophils *ex vivo* (Diep et al., 2010). Therefore, it was believed that rabbits might serve as a better animal model to study the role of PVL in *S. aureus* pathogenesis *in vivo*. Studies using rabbits as an animal model have shown contradicting to modest PVL-mediated effect in skin infections (Kobayashi et al., 2011; Lipinska et al., 2011), necrotizing pneumonia (Diep et al., 2010) and early stages of bacteremia (Diep et al., 2008). However, most rabbits are likely to have been previously exposed to *S. aureus* (Vancraeynest et al., 2006), resulting in the presence of antibodies against *S. aureus* that could influence the adequate assessment of leukocidins and other *S. aureus* virulence factors. Nevertheless, a recent study shows that active immunization with attenuated Hla and PVL prevents lethal development of necrotizing pneumonia in rabbits following challenge with a USA300 strain (Tran et al., 2020). The lack of an adequate *in vivo* model additionally hinders the assessment of the apparent redundant deployment of two leukocidins that target the same human C5aR1 (Spaan et al., 2013a, 2014, 2015b). The overlapping cell-tropism between leukocidins also remains enigmatic. All in all, the human specific nature of leukocidins has hindered the full assessment and contribution of leukocidins in *S. aureus* pathogenesis (Spaan et al., 2017). In addition, animals such as mice have been used as preclinical models in *S. aureus* vaccine and drug development, possibly resulting in the neglection of potential relevant targets in humans. The development of humanized mice, either via the engraftment of human hematopoietic stem cells (Knop et al., 2015) or the transgenic expression of human proteins (Pishchany et al., 2010), offers an alternative to investigate human tropic factors such as leukocidins *in vivo*.

### Studying Leukocidins *in vivo* and Controversy

Studies previously addressing leukocidins all suggested a role for leukocidins during systemic infection (Alonzo et al., 2013; Reyes-Robles et al., 2013; Spaan et al., 2014). HlgCB is present in 99% of *S. aureus* strains and is present in the *S. aureus* core genome (von Eiff et al., 2004; Alonzo and Torres, 2014). In contrast, PVL (*lukSF* gene) is located on the prophage locus ΦSa2 (Kaneko et al., 1998; Kaneko and Kamio, 2004; Otto, 2010) and only present in 2–3% of *S. aureus* isolates (Alonzo and Torres, 2014). A previous study using a rabbit model of necrotizing pneumonia has shown a PVL mediated phenotype *in vivo* (Diep et al., 2010), however, an epidemiological correlation between PVL and necrotizing pneumonia remains weak and controversial (Shallcross et al., 2012). A recent study showed that HlgCB contributes to increased bacterial loads in multiple organs beyond the primary infection site in hC5aR1^KI^ mice (Tromp et al., 2018). Even though both HlgCB and PVL target human C5aR1 *in vitro*, no PVL mediated phenotype was observed in a skin infection model using hC5aR1^KI^ mice (Tromp et al., 2018). This was contrary to previous findings that showed a PVL-mediated increase in skin lesion size when injected subcutaneously in (NOD)/severe combined immune deficiency (SCID)/IL2rγ^null^ (NSG) mice engrafted with human CD34^+^ umbilical cord blood cells (Tseng et al., 2015). Surprisingly, they did not observe an increase in CFU compared to the PVL^–^ *S. aureus* strains used in their study. A lack of PVL contribution in both systemic and skin infection in the hC5aR1^KI^ mice was attributed to an improper cellular susceptibility, as murine hC5aR^KI^ neutrophils were more resistant to PVL compared to HlgCB (Tromp et al., 2018). Based on chromatography elution profiles, individual leukocidin subunits are designated as S(slow)- or F(fast)-migrating component, and each having a molecular weight of approximately 33kD (Spaan et al., 2017). Non-cognate pairing of other leukocidin S- and F-subunits have been described *in vitro* (Prevost et al., 1995; Yoong and Torres, 2015; Koop et al., 2017; Spaan et al., 2017). Subsequent hybrid pairing of S-and F-subunits of HlgCB and PVL showed a LukF-PV mediated modulation of hC5aR^KI^ murine neutrophils, but not human neutrophils, to PVL sensitivity. These findings were suggestive of a yet uncharacterized target employed by LukF-PV to engage human target cells, possibly exposing a novel role for all leukocidin F-components. *In vivo* studies have additionally shown that non-cognate pairing of PVL and LukED is likely possible, but results in attenuated virulence of *S. aureus* (Yoong and Torres, 2015). As many clinical *S. aureus* isolates secrete all five bi-component toxins, non-cognate pairing of leukocidins results in a variety of toxin complexes (Spaan et al., 2017). The discovery of an F-component target, and possibly the existence of other F-component targets, might suggest a far more complex range of cytotoxic activities by employing non-cognate pairing, and possibly contributing to *S. aureus* pathogenesis. However, it remains to be resolved whether non-cognate pairing between leukocidins could also enhance *S. aureus* virulence *in vivo*. To further complicate things, humans carry pre-existing or develop antibodies against S- and F-components of leukocidins upon infection (Adhikari et al., 2015; Spaan et al., 2017). Due to the high homology between these components, the antibodies cross-react and cross-neutralize toxicity (Diep et al., 2016; Tran et al., 2020). This, in in conjunction with the likely non-canonical combination, complicates the *in vivo* determination of the exact role of each independent toxin.

### The Identification of the First Leukocidin F-Component Target

LukS-PV binds human neutrophils, monocytes but not lymphocytes (Colin et al., 1994; Jayasinghe and Bayley, 2005; Spaan et al., 2013a). LukS-PV, as a single subunit, is non-toxic and able to functionally inhibit C5a mediated activation of human neutrophils *in vitro* (Spaan et al., 2013a). It is not clear whether single PVL subunits contribute to the pathogenesis of *S. aureus*. It was long doubted whether leukocidins also employ F-components cellular surface targets as part of the initial interaction preceding pore-formation. HlgB, for example, can bind independent of HlgA to human erythrocytes (Ozawa et al., 1995), suggesting that a possible primary interaction of the S-component is not necessary in HlgAB mediated pore formation. In addition, a recent study identified an equine specific leukocidin, LukPQ, that showed an F-component mediated host-specific interaction when non-canonically paired with LukED (Koop et al., 2017). Until recently, the existence of F-component targets and the role of F-components in leukocidin pore formation was enigmatic. Following the lack of a PVL phenotype in a hC5aR^KI^ mouse model, a genome-wide CRISPR-based approach was used and CD45 was identified as the extracellular target for LukF-PV (Tromp et al., 2018). The pan-leukocyte marker CD45 is a highly conserved transmembrane glycoprotein and abundantly expressed on all nucleated hematopoietic cells (Charbonneau et al., 1988; Okumura et al., 1996; Hermiston et al., 2003). Follow up experiments showed that PVL specifically employs human CD45, and not murine CD45, as the target for LukF-PV. Even though the human CD45 specific interaction of LukF-PV likely explains the lack of a PVL mediated phenotype in hC5aR^KI^ mouse model (Tromp et al., 2018), the question arises if and how this additional LukF-PV binding to human CD45 prerequisite affects *S. aureus* pathophysiology.

### PVL and Necrotizing Pneumonia

In general, human *S. aureus* isolates scarcely carry the *lukSF* genes. However, *lukSF* carrying MRSA strains are suggested to be more virulent and have been associated with the development of severe necrotizing pneumonia (Lina et al., 1999; Gillet et al., 2002). *S. aureus* induced necrotizing pneumonia is suggested to be PVL, as well as neutrophil mediated (Diep et al., 2010). Nonetheless, the mechanisms involved in the onset of necrotizing pneumonia and the inducement of tissue necrosis are incompletely understood. It is possible that *S. aureus* gains access to the alveoli and induces the activation of neutrophils and the release pro-inflammatory mediators, resulting in the recruitment of neutrophils to the infected lung tissue (Hensler et al., 1994; Konig et al., 1995, 1997; Diep et al., 2010). It is further suggested that neutrophils are subsequently lysed by PVL, releasing proteases and ROS into the surrounding environment, inducing tissue damage of alveolar epithelial and endothelial barriers (Konig et al., 1995). C5aR1 was initially thought to be expressed exclusively on cells of myeloid origin. However, studies have shown the expression of C5aR1 on cells of solid organs such as vascular smooth muscle, lung bronchial and alveolar epithelial cells (Haviland et al., 1995). These cells of non-myeloid origin lack CD45 expression. It is not clear if PVL directly interacts or lyse lung cells via C5aR1 or whether the relative low expression level of C5aR1on these cells is sufficient to induce toxin-mediated pore-formation (Niemann et al., 2012; Perret et al., 2012).

### Novel Host-Factors Involved in Leukocidin-Receptor Interaction

Post-translational modification (PTM) of proteins is essential in many cellular processes (Walsh et al., 2005). In fact, PTM of GPCRs is important for regulating structure, function and association with natural ligands (Preobrazhensky et al., 2000; Farzan et al., 2001; Ulloa-Aguirre et al., 2006, 2014). However, PTM moieties on GPCRs have also been suggested to be involved in mediating the interaction with different human pathogens (Choe et al., 2005; Park et al., 2017). The sialic acid-binding adhesin (SabA) secreted by *Helicobacter pylori* mediates adherence specifically to the Lewis blood group antigens sLeX(Mahdavi et al., 2002). In addition, sLeX has been described as a PTM mediating affinity between many bacterial toxins and their targets. The *Escherichia coli* subtilase cytotoxin (SubAB), *S. enterica* typhoid toxin and *S. pneumoniae* cytolysin all interact in an sLeX dependent manner(Poole et al., 2018). A recent study also shows the identification of sialylation as a host-dependent-factor contributing to PVL, HlgCB, LukED and HlgAB mediated pore formation (Tromp et al., 2020). They suggest that the sialylation of C5aR1 and CXCR2 are not essential for PVL, HlgCB, HlgAB and LukED, but rather enhance the interaction and sensitivity to these toxins. The binding of LukS-PV to the N-terminus of C5aR1 was previously shown to be mediated by sulfation of the receptor using an N-terminal peptide (Spaan et al., 2013a). A more recent study confirms that the sulfation of the hC5aR1 is also not essential for PVL and HlgCB cytotoxicity, but rather enhances the interaction and sensitivity to these toxins (Tromp et al., 2020).

## Discussion: Our Unsettled Understanding of Leukocidins

It was long questioned why *S. aureus* would secrete multiple bi-component toxins that lyse phagocytes, a seemingly redundant strategy deployed by *S. aureus* to evade the host innate immune system. The first hints indicating that the secretion of multiple leukocidins is more than just simple redundancy came with the discovery that CCR5 is one of the receptors for LukED and therefore LukED could also lyse cells of the adaptive immunity (Alonzo et al., 2013). The discovery of other specific leukocidin GPCR targets further showed that leukocidins are not just a redundant feature, but possess the ability to select and drive host- and cell-specific cytotoxicity. However, that could not explain the apparent redundant deployment of two leukocidins that target the same human C5aR1 (Spaan et al., 2013a, 2014, 2015b). HlgCB and PVL both target neutrophils via C5aR1. On a molecular level however, the interaction between HlgCB and PVL with C5aR1 differs (Spaan et al., 2015b). While PVL exclusively interacts with the human and rabbit C5aR1, HlgCB interacts with multiple mammalian orthologs of C5aR1 and can additionally employ the human C3a receptor (hC3aR) as a target (Spaan et al., 2015b). Nonetheless, the interaction between HlgCB and hC3aR is inefficient (Spaan et al., 2015b).

**FIGURE 1:**
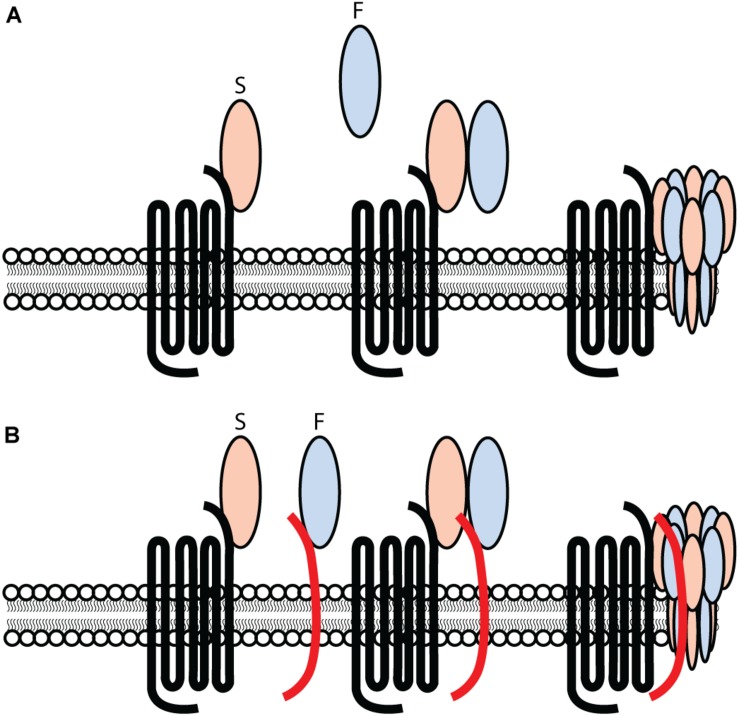
Challenging the previous model for PVL-cell interaction and pore-formation. **(A)** PVL consisting out of an S- and F-component, LukS-PV and LukF-PV respectively, interacting with hC5aR1 on target cells and inducing pore-formation in a stepwise approach. The primary interaction consists out of the S-component LukS-PV interacting with hC5aR1 on the surface of target cells. Subsequently, the F-component LukF-PV is recruited and interacts with the LukS-PV-hC5aR1 complex, consequently forming a ring-like octamer of alternating S- and F-components. A subsequent conformational change of the S- and F-components induces the inward collapse of the stem region forming a β-barrel pore that spans the cell membrane, consequently lysing the target cell. **(B)** PVL consisting out of an S- and F-component, LukS-PV and LukF-PV respectively. Two separate and independent interactions occur and consists of LukS-PV interacting with hC5aR1, and LukF-PV interacting with hCD45 on the surface of target cells. The interaction of LukS-PV and LukF-PV results in a complex inducing the formation of a ring-like octamer of alternating S- and F-components. A conformational change of the S- and F-components induces the inward collapse of the stem region forming a β-barrel pore that spans the cell membrane, resulting in the lysis of the target cell.

In 2007, a model for leukocidin function on target cells was proposed. With the exception of elucidating specific proteinaceous targets for all leukocidin S-subunits, the model for leukocidin pore-formation remained unchallenged. Current findings suggest a different approach to leukocidin pore-formation and cell specificity for PVL. The previous model (Figure 1A) suggested that leukocidins are secreted as water-soluble monomeric subunits by *S. aureus*. In the case of PVL, LukS-PV recognizes hC5aR1 on the surface of target cells. This interaction between LukS-PV and human C5aR1 is subsequently followed by the recruitment of the LukF-PV, resulting in oligomerization of alternating S- and F-components. This model suggests a stepwise process involving the primary interaction of LukS-PV, as previous studies were unable to detect LukF-PV binding in the absence of a primary bound LukS-PV (Colin et al., 1994). However, the identification of human CD45 as target for LukF-PV (Tromp et al., 2018) challenges this previous model for leukocidin pore-formation (Figure 1A,B). LukF-PV can bind independent of LukS-PV to human CD45 expressing cells, indicating that the primary interaction of LukS-PV in not necessary for the recruitment of LukF-PV (Tromp et al., 2018). LukS-PV and LukF-PV can interact independent of each other. However, contrary to C5aR1, the expression of CD45 is not essential for PVL cytotoxicity, but rather enhances the interaction and sensitivity to C5aR1 expressing cells (Tromp et al., 2018). This is further enhanced by additional PTM moieties (Tromp et al., 2020). Tyrosylprotein Sulfotransferase 2 (TPST2) and 3′-phosphoadenosine 5′-phosphosulfate synthetase 1 (PAPSS1) were picked-up in a genome-wide screening for toxin resistance and are involved in the sulfation of C5aR1, enhancing the interaction with PVL and HlgCB. However, there is variation in expression levels of TPST amongst cell types (Farzan et al., 2002; Ludeman and Stone, 2014; Tan et al., 2014). Tyrosine sulfation is heterogeneous and tissue specific (Mishiro et al., 2006; Ludeman and Stone, 2014) resulting in variable sulfation profiles possibly contributing to the host and cellular tropism of leukocidins. It remains to be established whether there is variability in sulfation of GPCRs on phagocytes during different stages of activation or infection, and possibly contributing to the interaction of leukocidins with their respective receptor. Studies suggest that the PVL induced octameric pore consists of a LukS-PV/LukF-PV ratio of 1:1, in which each LukS-PV monomer binds one C5aR1 (Miles et al., 2002; Das et al., 2007; Haapasalo et al., 2018). It is unknown whether each LukF-PV is also bound to CD45.

The function of CD45 in innate immunity and bacterial infections has rarely been assessed and is unknown. As CD45 plays a role in T-cell development, signaling and function (Johnson et al., 2000; Hermiston et al., 2003), it is possible that *S. aureus* might modulate lymphocytes signaling via the binding of LukF-PV to CD45. In addition, as neutrophils are the primary target, *S. aureus* can designate the pan-leukocyte marker CD45, followed by C5aR1 as a phagocytic marker, forming part of a two-step-control mechanism (Tromp et al., 2018). This enables *S. aureus* to differentiate between the potentially non-threatening C5aR1 expressing cells and neutrophils. In this simple, but yet effective way of selecting CD45^+^C5aR1^+^ cells, *S. aureus* can specifically target neutrophils in an infection environment. However, assessing the contribution of human CD45 in PVL mediated *S. aureus* pathogenesis, or as a single LukF-PV subunit *in vivo*, would be challenging. The generation of a double hC5aR1/hCD45 knock-in mouse as a murine model might elucidate the importance of the PVL-CD45 interaction in infection. However, the process is laborious and the lack of an existing human CD45 knock-in mouse model impedes the development of a double hC5aR1/hCD45 knock-in mouse via backcross. This is not a surprise, as the gene encoding CD45 (*PTPRC*) is highly complex with 34 exons spanning over 110 kb on the genome, making it not amenable for CRISPR-mediated knock-in without a preliminary characterization of the exons involved in the LukF-hCD45 interaction (Okumura et al., 1996; Hermiston et al., 2003; Rheinlander et al., 2018). In addition, the expression of human CD45 may trigger a deleterious phenotype as CD45 is a phosphatase expressed on all nucleated cells of myeloid origin (Charbonneau et al., 1988; Kung et al., 2000; Hermiston et al., 2003; Rheinlander et al., 2018). Nevertheless, the identification of the F-component receptor for PVL, and intracellular processes refining leukocidin cytotoxicity, has consequences for our current understanding of leukocidin pore-formation. A better understanding of host-factors involved in leukocidin activity might reveal targets for anti-virulence strategies and improve models to study *S. aureus* pathophysiology. Nevertheless, studying *S. aureus* leukocidin remains a challenging endeavor.

## Author Contributions

AT and JS wrote the manuscript.

## Conflict of Interest

The authors declare that the research was conducted in the absence of any commercial or financial relationships that could be construed as a potential conflict of interest.
